# Supplementary material for: Benchmarking DNA isolation methods for marine metagenomics
Source: Sci Rep. 2023 Dec 13;13:22138. doi: 10.1038/s41598-023-48804-z (PMC10719357; doi:10.1038/s41598-023-48804-z)
Supplement: Supplementary file 1 — Supplementary Figures. [file 41598_2023_48804_MOESM1_ESM.docx]

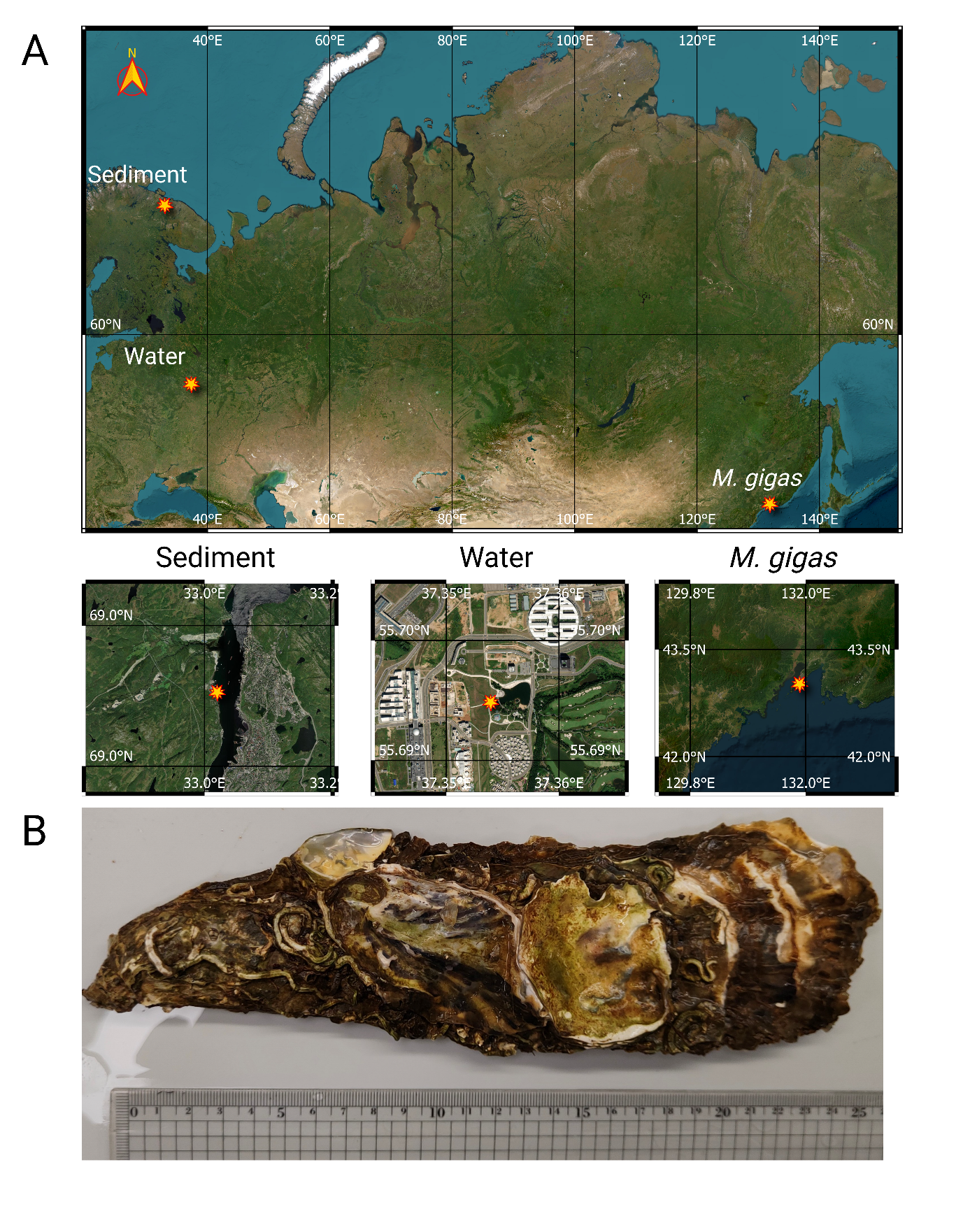


**Fig. S1**. **A)** Geographical locations for water, sea sediment, and Pacific oyster (*M. gigas*) collection spots. Map was prepared using Open Source Geospatial Foundation Project (http://qgis.org) **B)** Selected individual of Pacific oyster (*M. gigas*) aligned with a centimeter ruler.


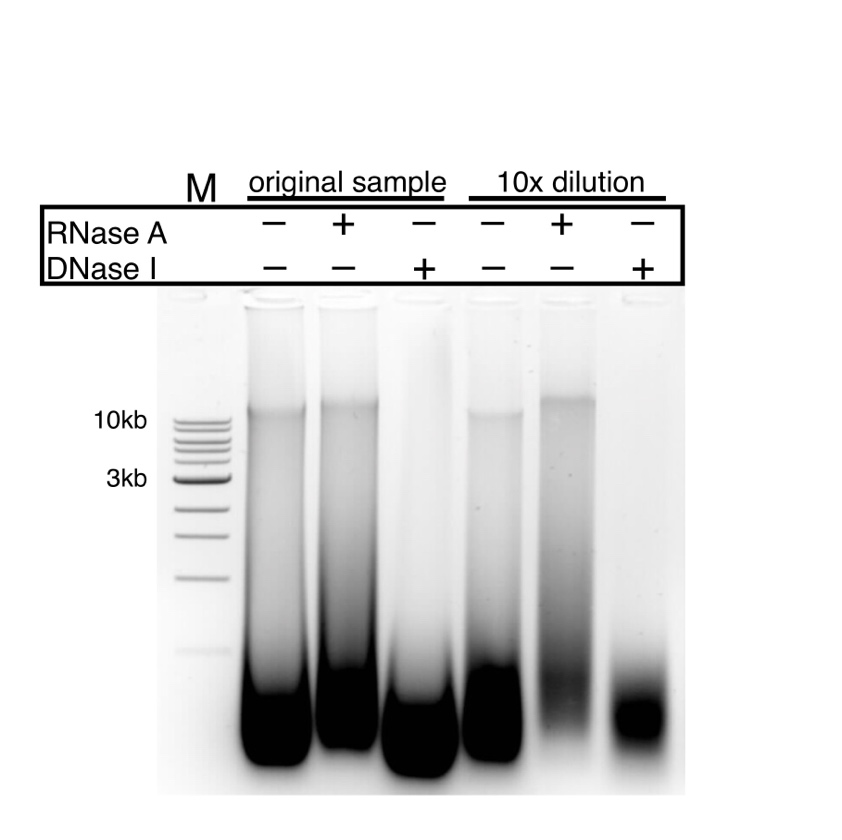


**Fig. S2.** DNase and RNase treatment of *M.gigas* DNA sample isolated with Stool kit.


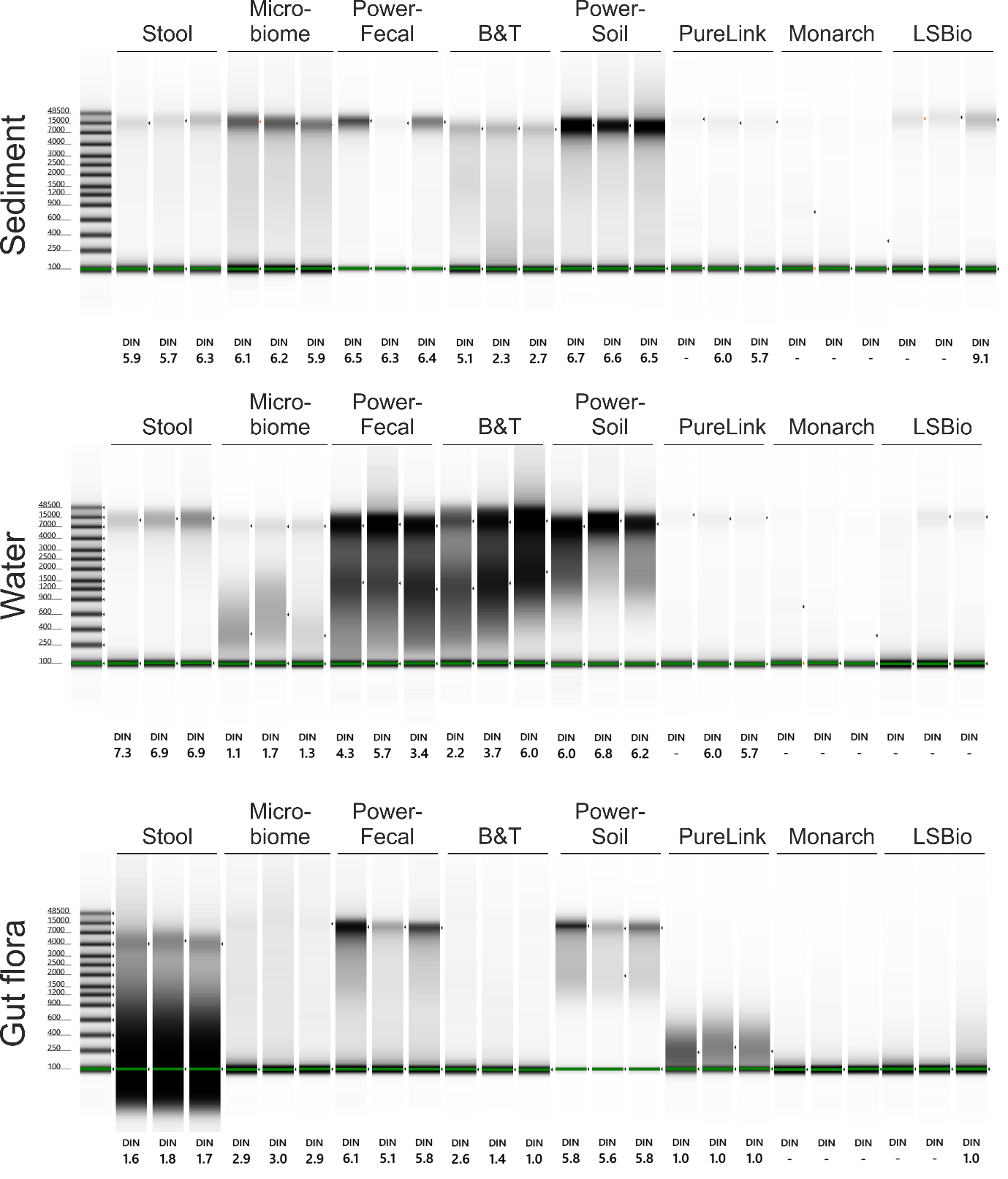


**Fig. S3.** Capillary electrophoresis estimation of the DIN values performed on a TapeStation 4150 (Agilent) with Genomic DNA ScreenTape System.


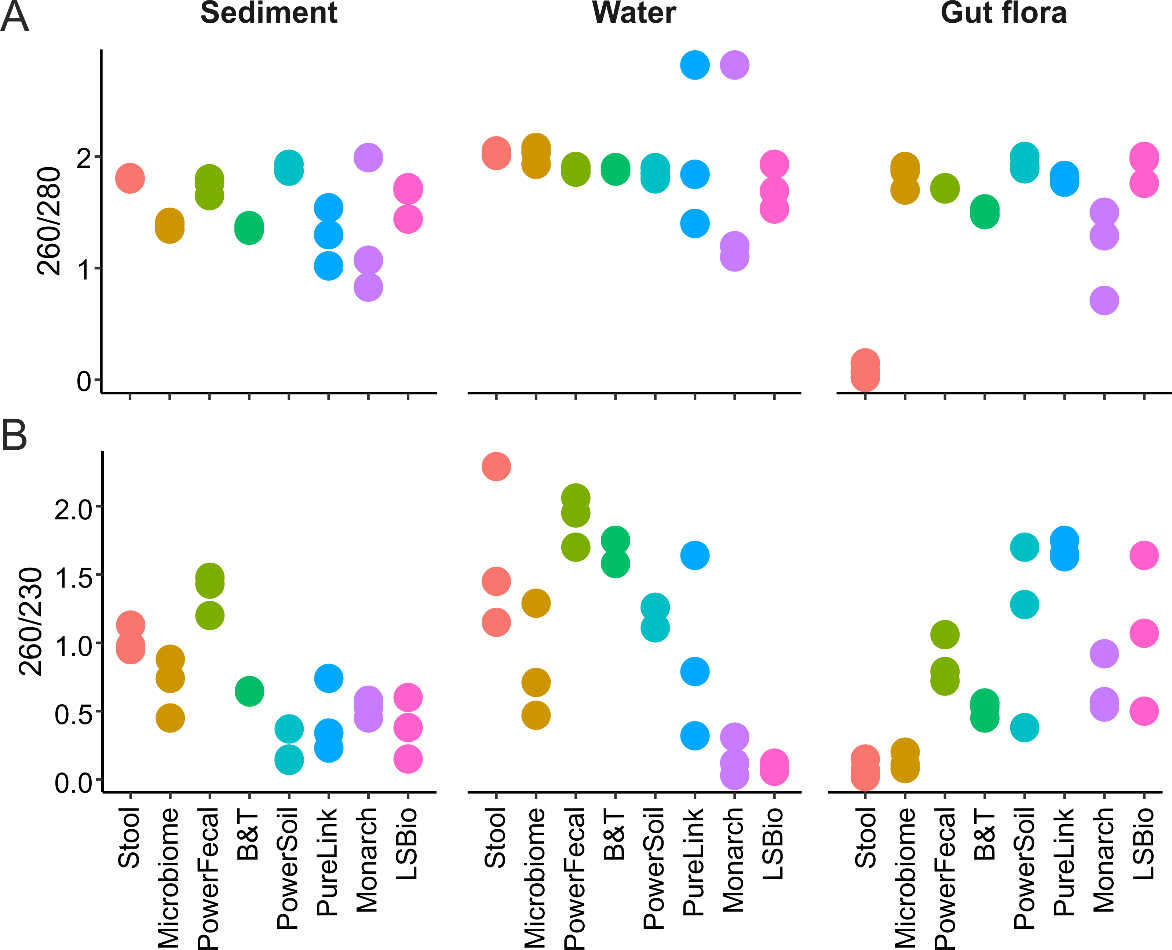


**Fig. S4.** DNA purity assessed by 260/280 (**A**) and 260/230 (**B**) absorption ratios. Data for three technical replicates are shown.


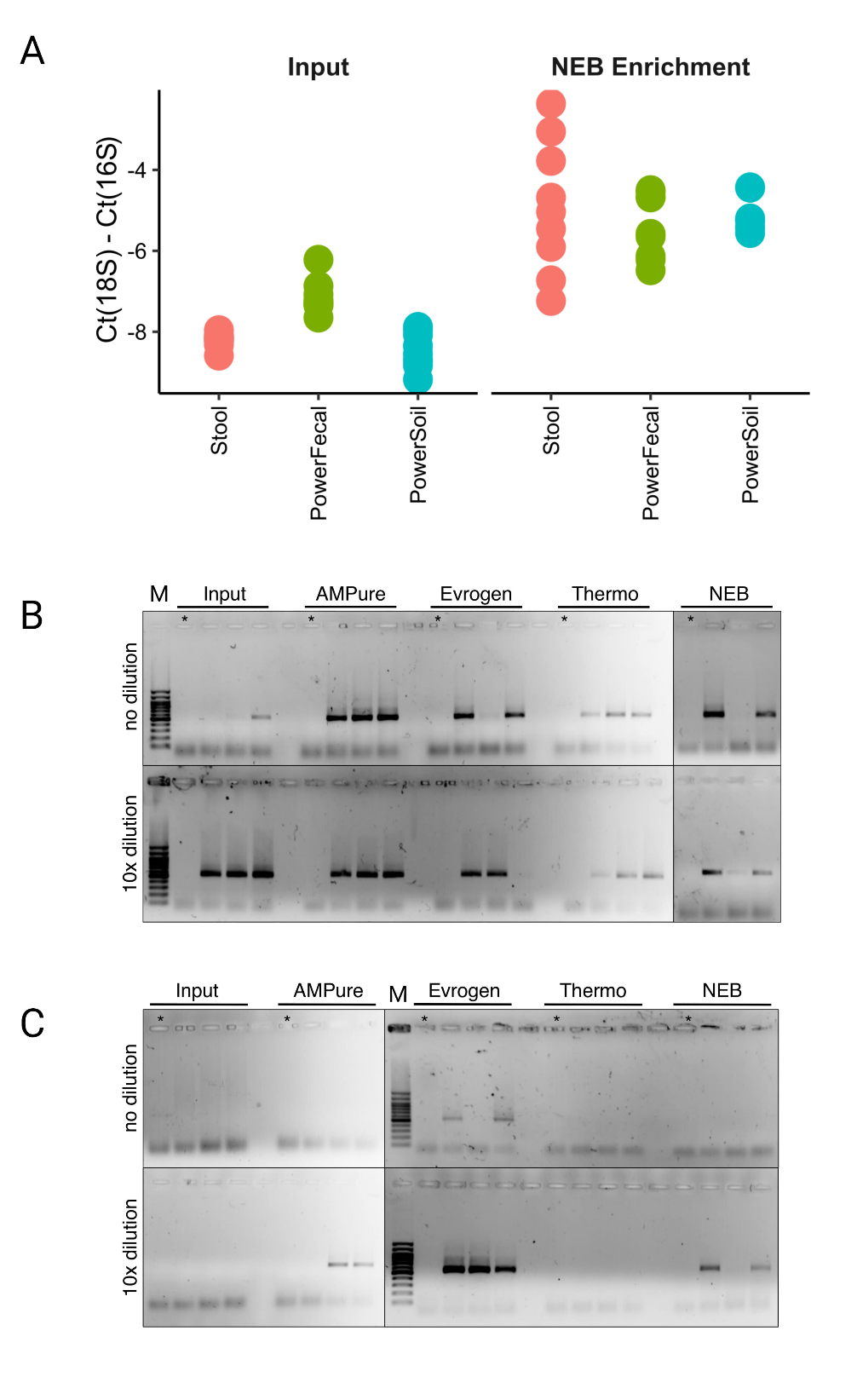


**Fig. S5.** (**A**) Retention of DNA relative to the input amount (50 ng for Stool or 150 ng for Microbiome kits) after additional re-purification procedures. (**B-C**) Results of the 16S rRNA gene PCR with sea sediment samples purified with Stool (**B**) and Microbiome (**C**) kits. PCR was performed with non-diluted and 10-fold diluted input DNA or DNA additionally re-purified with indicated kits (see Methods). *- no-input control that was re-purified in parallel with experimental samples to estimate potential contamination of the re-purification kit solutions with microbial DNA. M – 100 bp Plus GeneRuler DNA ladder (Thermo Scientific). Products were loaded on 3 agarose gels and run in parallel, fragments of different gels are separated by a black line. Uncropped gels can be found in Fig. S13.


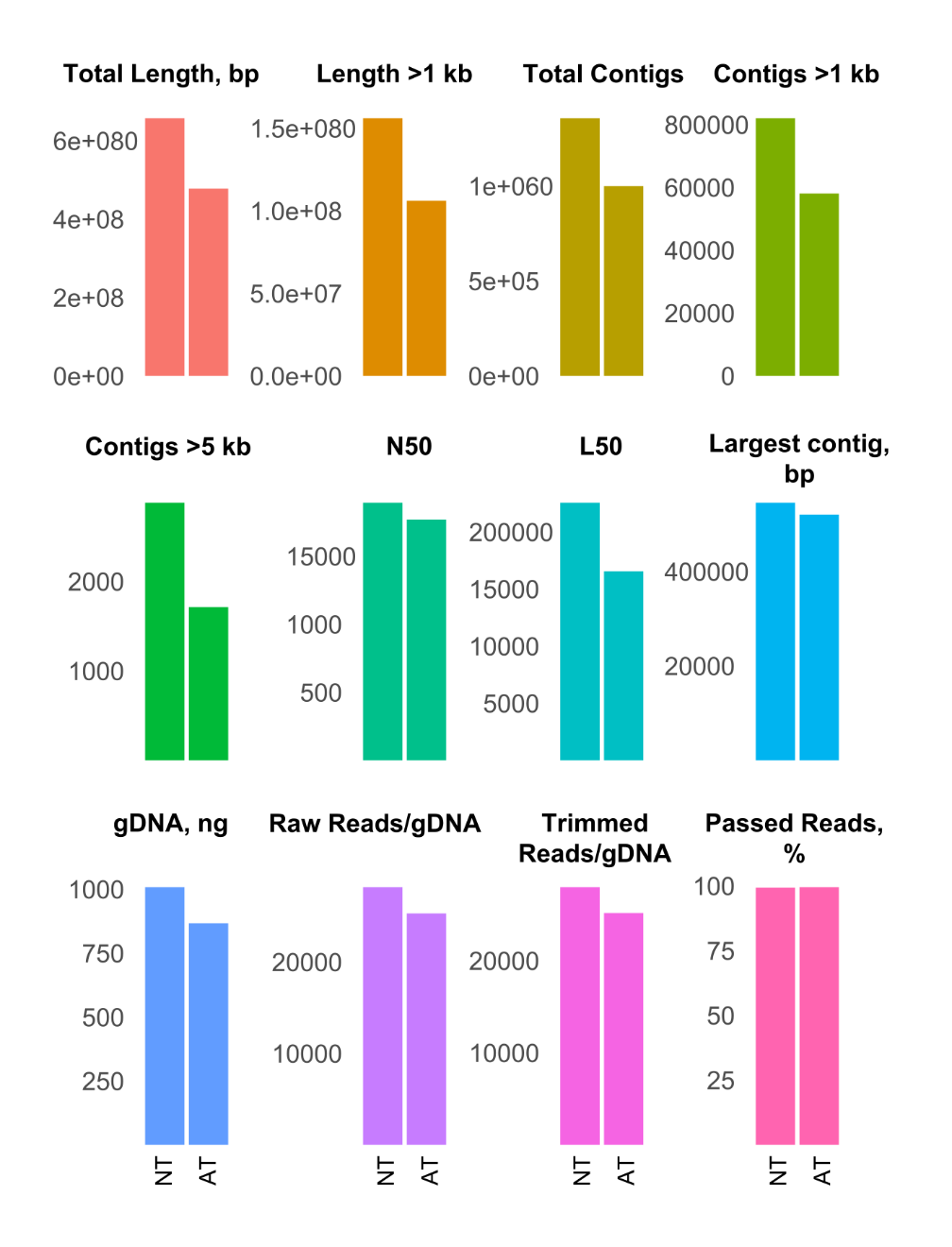


**Fig. S6.** Parameters of shotgun BGI libraries obtained from sediment sample purified with PowerFecal kit (NT – no treatment) and additionally re-purified with Evrogen column kit (AT - additional treatment).


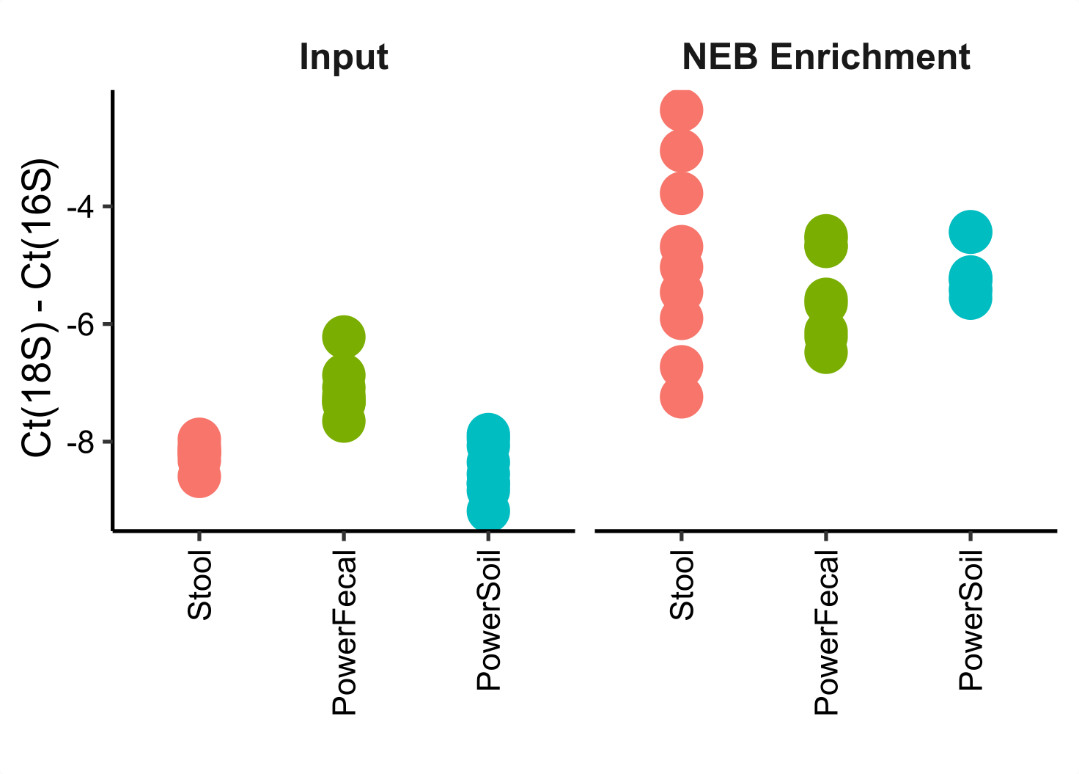


**Fig. S7.** qPCR Ct values obtained with 18S and 16S rRNA gene-specific primers with the input and NEB Enrichment re-purified DNA samples. Data for three technical qPCR replicates for each of the three kit purification replicates are shown.


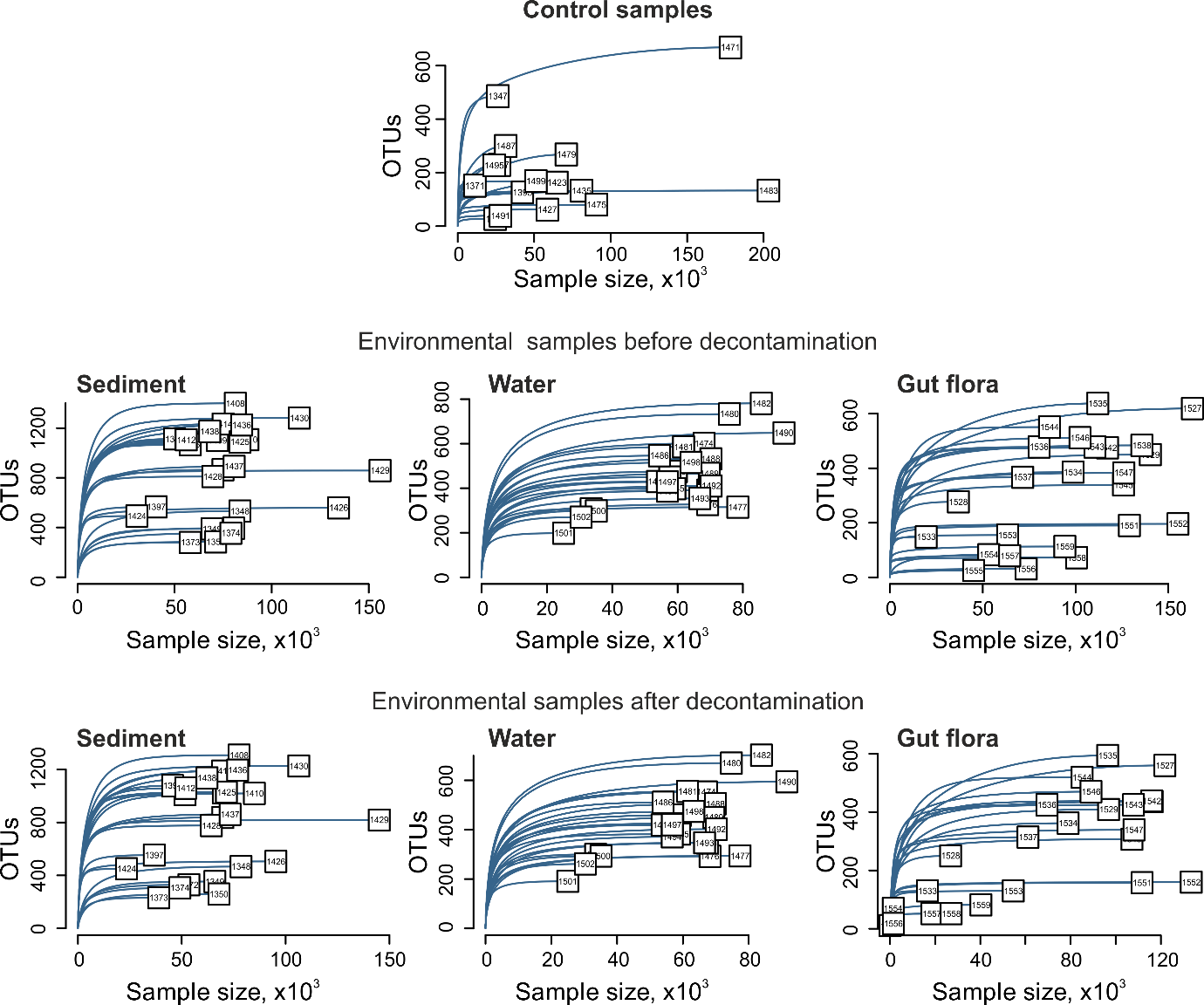


**Fig. S8**. Rarefaction curves for control samples (upper row), natural samples before decontamination (middle), and natural samples after decontamination (bottom row).


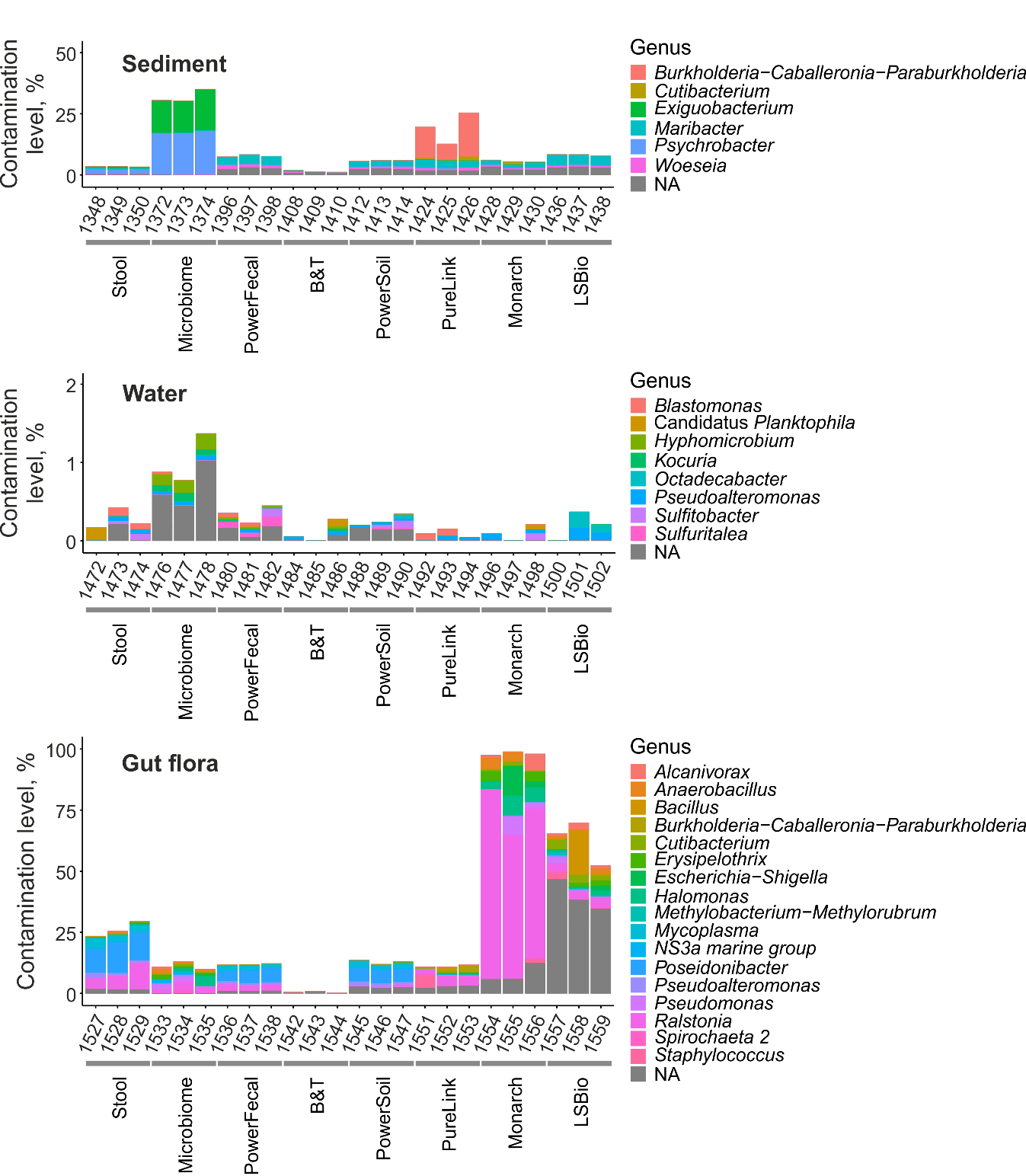


**Fig. S9**. Contamination levels of natural samples. Data is shown for all technical replicates independently. Genera with relative abundances >1% are shown.


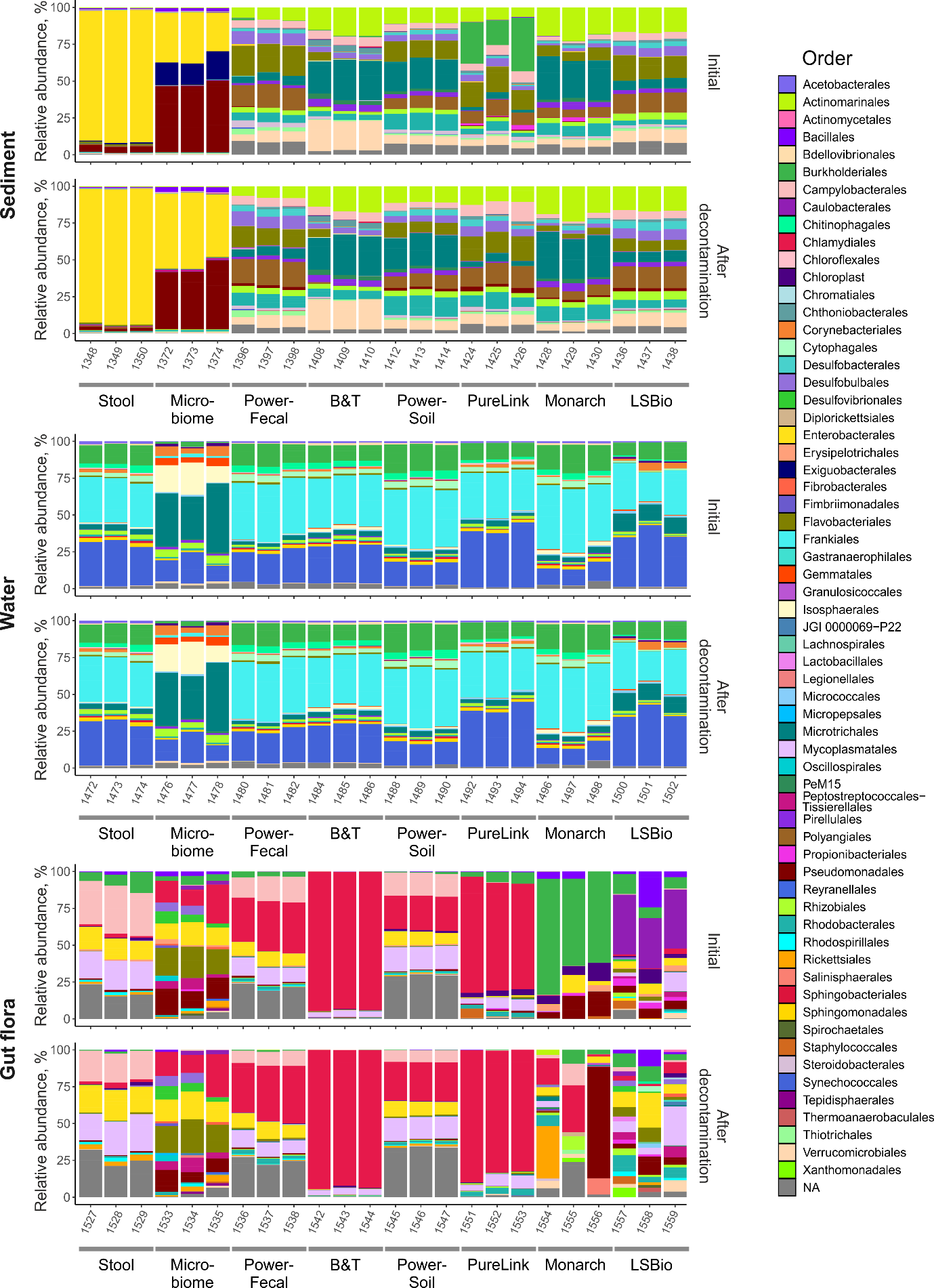


**Fig. S10**. Microbial communities’ composition of natural samples before decontamination (Initial) and after the decontamination on an order level. Data is shown for all technical replicates independently. Orders with relative abundances >1% are shown.


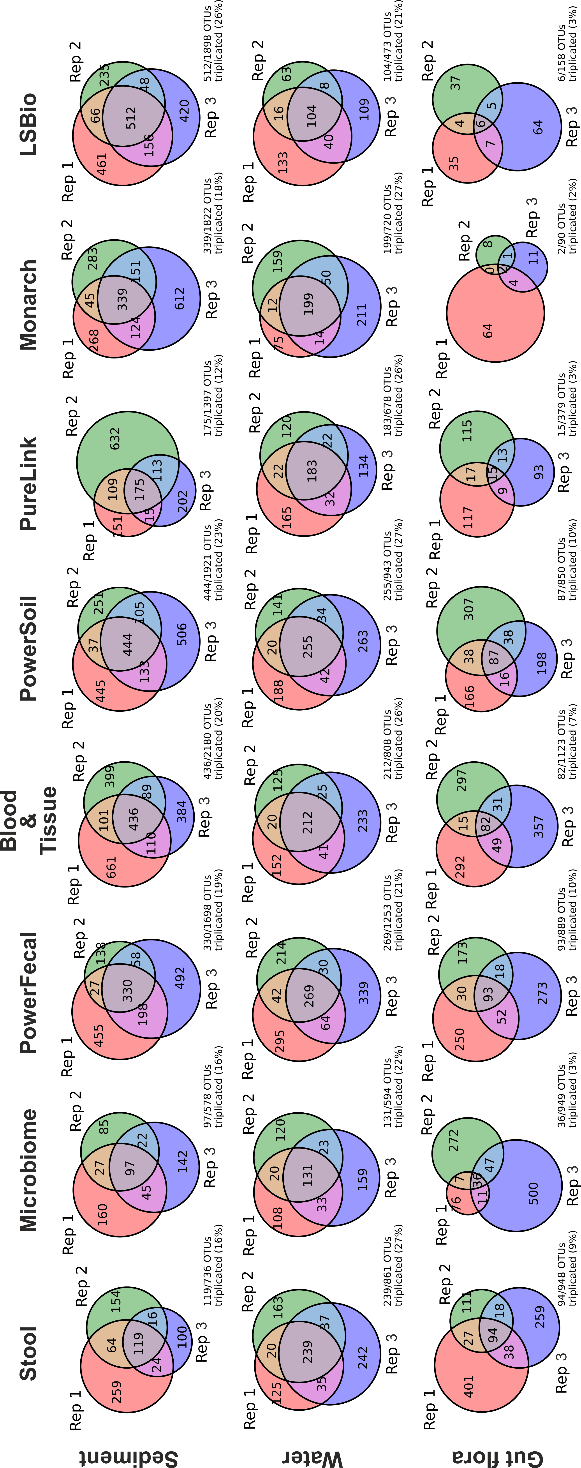


**Fig. S11**. **Reproducibility of DNA-extraction kits**. Venn diagrams representing the intersections of lists of non-zero OTUs (OTUs with a non-zero abundance) for three technical replicates obtained with specified DNA-extraction kits. Below the diagram, reproducibility level is shown (%) as a fraction of non-zero OTUs found in all three replicates from the total number of unique non-zero OTUs found in at least one replicate.


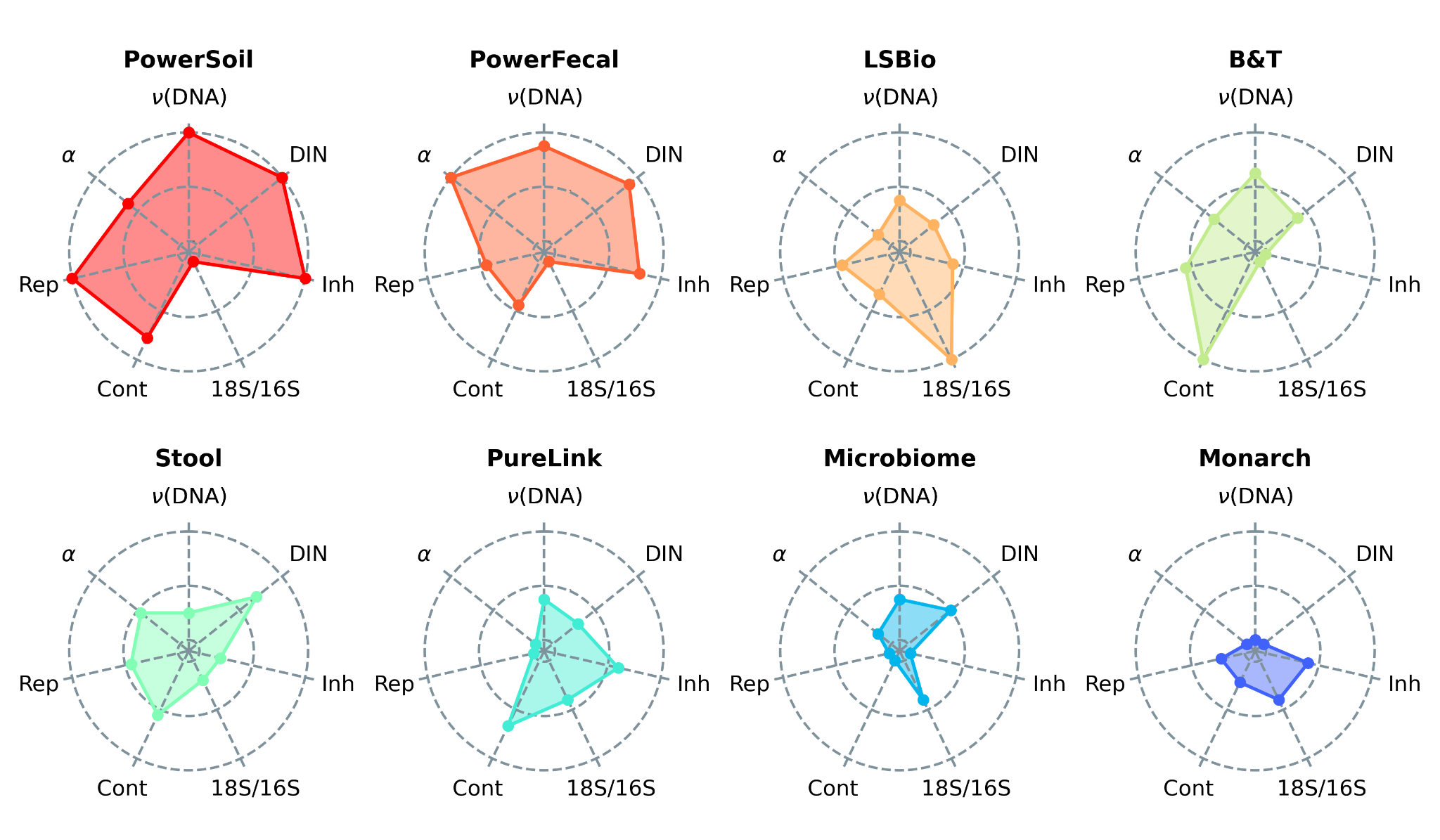


**Fig. S12**. Radar-plots demonstrating the average performance of DNA-extraction kits. 𝜈(DNA) - DNA yield, DIN - DNA integrity, Inh - presence of PCR inhibitors (higher rank indicates the lower level of inhibitors), 18S/16S - 18S/16S ratio (higher rank indicates the lower ratio), Cont - contamination level (higher rank indicates the lower level of contamination), Rep - reproducibility level, 𝛼 - alpha-diversity. Kits were ordered by the sum of ranks.


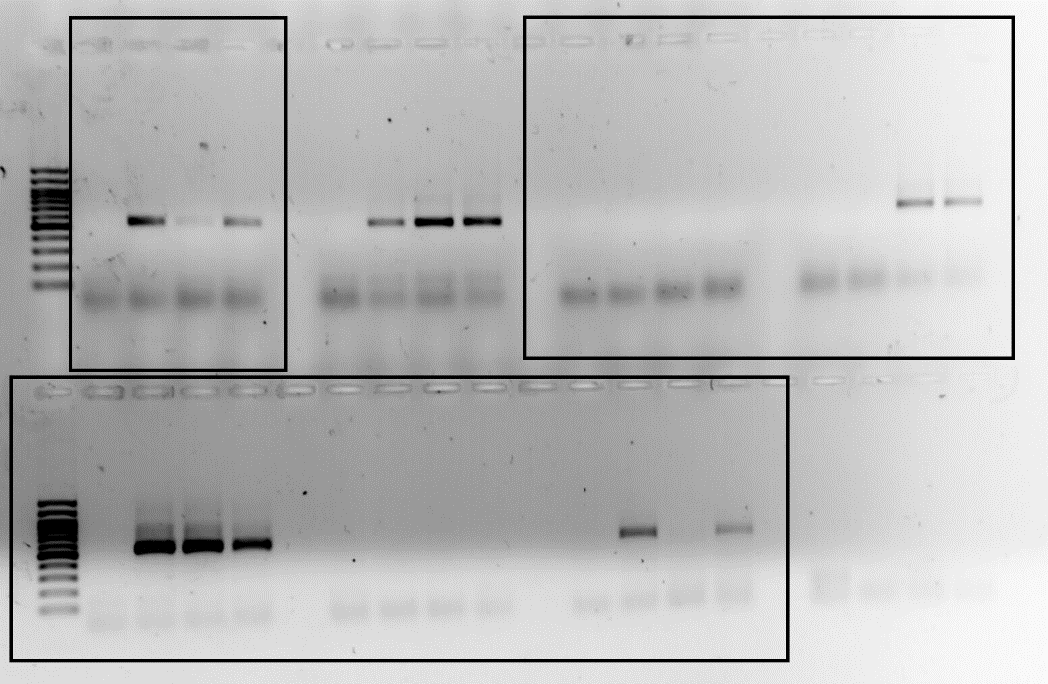


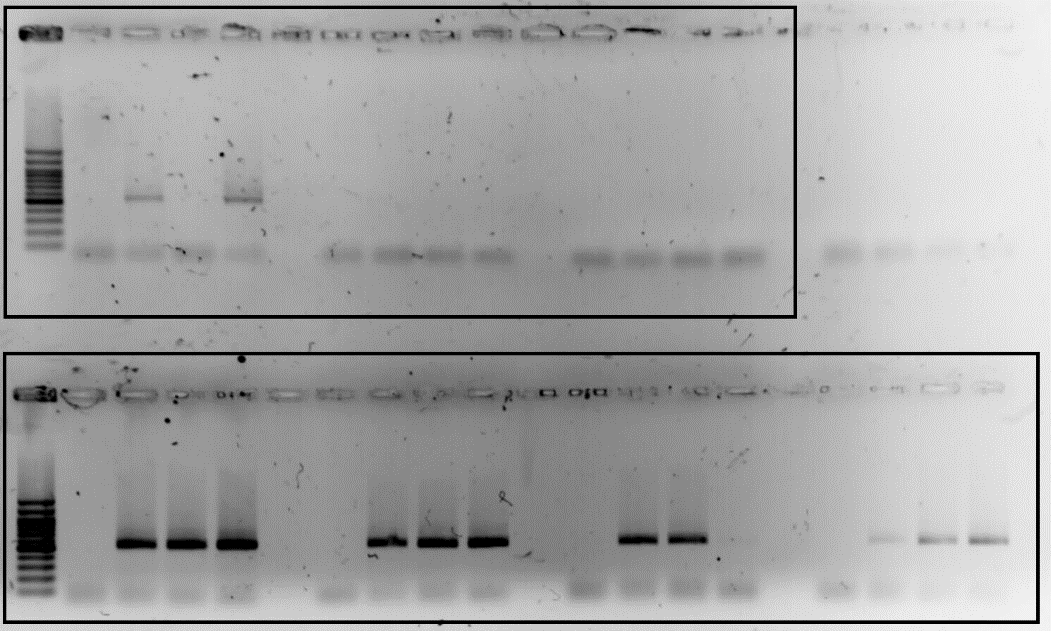


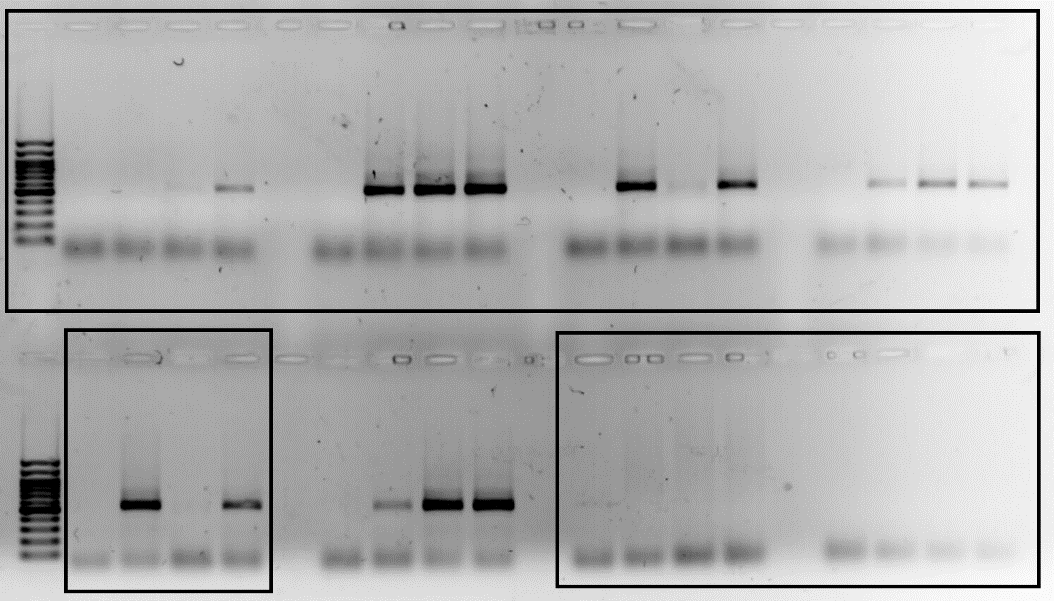


**Fig. S13.** Uncropped agarose gels used to generate Fig. S5B,C. Cropped fragments used to build Fig. S5B,C are shown in frames.
